# Supplementary material for: ProteinShader: illustrative rendering of macromolecules
Source: BMC Struct Biol. 2009 Mar 30;9:19. doi: 10.1186/1472-6807-9-19 (PMC2672931; doi:10.1186/1472-6807-9-19)
Supplement: Additional file 1 — ProteinShader program without source code. This compressed file contains the complete ProteinShader program including associated libraries, but no source code. A README.txt file gives an overview of the ProteinShader distribution, and the index.html file in the help subdirectory has directions on getting started with the program as well as a set of tutorials. [file 1472-6807-9-19-S1.zip › ProteinShader-beta-0_9_4-binary/help/api/org/proteinshader/graphics/typography/class-use/GlyphSet.html]

Uses of Class org.proteinshader.graphics.typography.GlyphSet (ProteinShader API)


|  |  |  |
| --- | --- | --- |
| **Overview** \| **Package** \| **Class** \| **Use** \| **Tree** \| **Deprecated** \| **Index** \| **Help** | |  |
| PREV   NEXT | **FRAMES**    **NO FRAMES**     **All Classes** |  |


---


## **Uses of Class org.proteinshader.graphics.typography.GlyphSet**

| Packages that use GlyphSet | |
| --- | --- |
| **org.proteinshader.graphics.typography** | Holds the classes needed for applying text labels to the curved surfaces of tubes and ribbons. |

Uses of GlyphSet in org.proteinshader.graphics.typography

| Methods in org.proteinshader.graphics.typography that return GlyphSet | |
| --- | --- |
| `GlyphSet` | `GlyphSetFactory.readGlyphSet(Properties p)`             Reads information from a source and constructs a GlyphSet object. |
| `GlyphSet` | `GLF2GlyphSetFactory.readGlyphSet(Properties p)`             Reads information from a '.glf' file and constructs a GlyphSet object. |

---


|  |  |  |
| --- | --- | --- |
| **Overview** \| **Package** \| **Class** \| **Use** \| **Tree** \| **Deprecated** \| **Index** \| **Help** | |  |
| PREV   NEXT | **FRAMES**    **NO FRAMES**     **All Classes** |  |


---

# *Copyright © 2007-2008*
